# Contribution of Adipose Tissue to the Chronic Immune Activation and Inflammation Associated With HIV Infection and Its Treatment

**DOI:** 10.3389/fimmu.2021.670566

**Published:** 2021-06-18

**Authors:** Christine Bourgeois, Jennifer Gorwood, Anaelle Olivo, Laura Le Pelletier, Jacqueline Capeau, Olivier Lambotte, Véronique Béréziat, Claire Lagathu

**Affiliations:** ^1^ CEA - Université Paris Saclay - INSERM U1184, Center for Immunology of Viral Infections and Autoimmune Diseases, IDMIT Department, IBFJ, Fontenay-aux-Roses, France; ^2^ Sorbonne Université, INSERM UMR_S 938, Centre de Recherche Saint-Antoine, Institut Hospitalo-Universitaire de Cardio-métabolisme et Nutrition (ICAN), FRM EQU201903007868, Paris, France; ^3^ AP-HP, Groupe Hospitalier Universitaire Paris Saclay, Hôpital Bicêtre, Service de Médecine Interne et Immunologie Clinique, Le Kremlin-Bicêtre, France

**Keywords:** adipose tissue, HIV infection, fat, antiretroviral treatment, chronic inflammation, chronic immune activation

## Abstract

White adipose tissue (AT) contributes significantly to inflammation – especially in the context of obesity. Several of AT’s intrinsic features favor its key role in local and systemic inflammation: (i) large distribution throughout the body, (ii) major endocrine activity, and (iii) presence of metabolic and immune cells in close proximity. In obesity, the concomitant pro-inflammatory signals produced by immune cells, adipocytes and adipose stem cells help to drive local inflammation in a vicious circle. Although the secretion of adipokines by AT is a prime contributor to systemic inflammation, the lipotoxicity associated with AT dysfunction might also be involved and could affect distant organs. In HIV-infected patients, the AT is targeted by both HIV infection and antiretroviral therapy (ART). During the primary phase of infection, the virus targets AT directly (by infecting AT CD4 T cells) and indirectly (via viral protein release, inflammatory signals, and gut disruption). The initiation of ART drastically changes the picture: ART reduces viral load, restores (at least partially) the CD4 T cell count, and dampens inflammatory processes on the whole-body level but also within the AT. However, ART induces AT dysfunction and metabolic side effects, which are highly dependent on the individual molecules and the combination used. First generation thymidine reverse transcriptase inhibitors predominantly target mitochondrial DNA and induce oxidative stress and adipocyte death. Protease inhibitors predominantly affect metabolic pathways (affecting adipogenesis and adipocyte homeostasis) resulting in insulin resistance. Recently marketed integrase strand transfer inhibitors induce both adipocyte adipogenesis, hypertrophy and fibrosis. It is challenging to distinguish between the respective effects of viral persistence, persistent immune defects and ART toxicity on the inflammatory profile present in ART-controlled HIV-infected patients. The host metabolic status, the size of the pre-established viral reservoir, the quality of the immune restoration, and the natural ageing with associated comorbidities may mitigate and/or reinforce the contribution of antiretrovirals (ARVs) toxicity to the development of low-grade inflammation in HIV-infected patients. Protecting AT functions appears highly relevant in ART-controlled HIV-infected patients. It requires lifestyle habits improvement in the absence of effective anti-inflammatory treatment. Besides, reducing ART toxicities remains a crucial therapeutic goal.

## The Biology of Adipose Tissue

### Several “Colors” of Adipose Tissue: White, Brown, Beige, and Pink

Adipose tissue (AT) is a loose connective tissue composed of differentiated adipocytes and stroma/vascular cells (a heterogeneous cell population including endothelial cells, immune cells, fibroblasts, and adipocyte precursors). White adipocytes are characterized by a single, large droplet of cytoplasmic fat and a flattened nucleus. Brown adipocytes contain several small lipid droplets and large numbers of mitochondria and lysosomes; the latter are responsible for the tissue’s brown color. Beige adipocytes (white adipocytes that have differentiated into brown adipocytes) are characterized by multilocular lipid droplets. Lastly, pink adipocytes are white adipocytes that have differentiated into milk-producing gland cells during pregnancy, lactation, and post-lactation (1).

The various ATs differ with regard to their metabolic activity, sites, plasticity, vascularization and innervation. White adipose tissue (WAT) is the main site for lipid storage and mobilization and has a high secretion capacity. The activity of WAT is strongly linked to inflammation in healthy individuals (where low-grade inflammation is required for correct metabolic activity) (2), and even more so in ageing and in people with fat gain/obesity (where the more intense inflammation contributes to the loss of metabolic activity) (3). Brown adipose tissue (BAT, mainly characterized in rodents) ensures non‐shivering thermogenesis; it can maintain thermal homeostasis by dissipating large amounts of energy in the form of heat. It used to be thought that BAT was only present in meaningful amounts in infants and that it regressed and become metabolically inactive in adults. However, recent positron emission tomography (PET) imaging studies of glucose uptake have revealed the presence of substantial deposits of thermogenic fat in adult humans (4, 5). Besides being highly metabolically active, BAT is densely innervated by the sympathetic nervous system (6) and is relatively insensitive to inflammatory signals. Recent studies have demonstrated the presence of brown-like adipocytes in WAT. These “Beige” adipocytes (also referred to as “browned” or “brite” [“brown in white”] adipocytes) are highly thermogenic adipocytes (6). They are functionally flexible and can either store or dissipate energy, depending on the environmental or physiological circumstances. Like brown adipocytes, beige adipocytes oxidize fatty acids and glucose and have large numbers of mitochondria. In response to various stimuli, the energy is dissipated as heat through uncoupled respiration (7). This mechanism is mediated by the uncoupling protein-1 (UCP-1) present in the inner mitochondrial membrane. Beige adipocytes can enhance energy expenditure by inducing a futile cycle that involves free fatty acid (FFA) β-oxidation and re-esterification (8, 9). This process might constitute a crucial adaptive response to excess energy supplies. In humans, the correlation between leanness and high levels of brown and beige fat activity suggests that these ATs have an important metabolic role (4, 5, 10).

Adipocytes are highly plastic, as exemplified by the beiging process, which corresponds to recruitment of the adaptive thermogenic adipocytes. Beiging may be a novel therapeutic target for mitigating lipid storage, alleviating metabolic disorders and thus dampening the associated inflammatory profile.

### A Focus on WAT and Its Unique Properties

#### WAT Is Influential Because of Its Large Mass and Broad Distribution

WAT is the body’s main energy reservoir; it stores energy as triglycerides and releases energy (in response to hormonal stimuli) as FFAs. This AT accounts for 15 to 25% of body weight in lean, healthy individuals and much greater percentage in overweight and obese people. In contrast to BAT, WAT is distributed throughout the body. There are two main types of WAT: subcutaneous adipose tissue (SCAT, accounting for about 80% of AT in lean individuals) and visceral adipose tissue (VAT, located in the intra-abdominal cavity, e.g. at omental, mesenteric, and retroperitoneal sites). Although lean individuals have a small amount of VAT, this tissue expands considerably in people with metabolic disorders. Intra-abdominal VAT is able to communicate with nearby internal organs, such as the intestinal tract and the liver. Several mechanisms are involved in this communication, with the release of metabolites, hormones, cytokines and miRNAs (11, 12). Perivascular AT, epicardial AT, lymph-node (LN)-associated AT, bone-marrow (BM)-infiltrating AT, and (in the context of metabolic disorders) ectopic AT in liver or muscles have been described (see section 4). Lastly, the anatomical distribution of WAT in contact with the external environment (such as the intestinal tract and the skin) and with immune structures (LNs, BM, and thymus) might be crucial in the modulation of local immune responses.

#### WAT Is Also a Major Endocrine Organ

WAT is less metabolically active than BAT but has a unique endocrine profile. The main endocrine activity in WAT is adipokine secretion [for a review, see (13)]. In fact, WAT secretes over 400 different adipokines, which are usually categorized as being anti-inflammatory or pro-inflammatory but also contribute to metabolic regulation. Some of these adipokines are produced by the adipocytes themselves (such as the prototypical adipokines leptin and adiponectin), whereas others are mainly produced by immune cells located in the AT (interleukin (IL)-6, tumor necrosis factor (TNF)-α, IL-8, monocyte chemoattractant protein 1 (MCP-1), regulated upon activation, normal T cell expressed and presumably secreted chemokine (RANTES), *etc.*) (**Table 1**). The well-known satiety factor leptin is secreted specifically by adipocytes in SCAT and (to a lesser extent) VAT and has a role in energy regulation. Blood leptin levels are correlated with the AT mass. Adiponectin is the other main adipocyte-specific adipokine. It is mainly secreted by VAT and is known to enhance fatty acid oxidation by muscle tissue and to increase the action of insulin in the liver. Thus, blood adiponectin levels are inversely correlated with insulin resistance. Importantly, both leptin and adiponectin also have immunomodulatory functions (20, 21).

**Table 1:** Cytokine production by AT.

	Differences between VAT and SCAT			Cell source	
				Adipocytes	Stromal/vascular cells
**Pro-inflammatory factors**					
IL-6	VAT	>	SCAT	++	++
TNF-α	VAT	=	SCAT	+	+++
IL-1β	VAT	>	SCAT	+	++
Resistin	VAT	>	SCAT	+	++
Leptin	SCAT	>	VAT	+++	–
**Chemokines**					
IL-8	VAT	>	SCAT	+	++
RANTES	VAT	>	SCAT	+	++
MCP-1	VAT	>	SCAT	+	++
**Anti-inflammatory factors**					
IL-10	VAT	=	SCAT	+	++
IL-1Rα	VAT	=	SCAT	+	+
IL-4	VAT	>	SCAT	+	++
Adiponectin	VAT	>	SCAT	+++	–

The table gives a non-exhaustive list of adipokines/cytokines with prominent pro- or anti-inflammatory activities from among the 400 produced by AT. Three groups are defined here: pro-inflammatory cytokines (IL-6, Tumor necrosis factor (TNF-α), IL-1β, resistin, and leptin); chemokines (IL-8, monocyte chemoattractant protein 1 (MCP-1), regulated upon activation, normal T cell expressed and presumably secreted chemokine (RANTES)), and anti-inflammatory factors (IL-10, IL-1Rα, IL-4, and adiponectin). The secretion profile depends on the site (i.e. SCAT vs. VAT) and the cell type (14–19).

SCAT, Subcutaneous adipose tissue; VAT, Visceral adipose tissue; IL, Interleukin.

The influence of adipose-secreted factors on systemic and distal inflammation, angiogenesis, vascular homeostasis, fibrolysis, and the immune system is not fully understood (22) and is being evaluated in various diseases [including inflammatory arthritis, autoimmunity (23), inflammatory bowel disease (24), and cancer (25)]. The AT’s adipokine secretion profile is strongly modulated by the metabolic and inflammatory context. The various bioactive adipokines exert autocrine, paracrine and endocrine effects on local and systemic targets. The AT’s endocrine activity appears to depend on the adipokine in question and the tissue site: SCAT produces IL-6 and TNF-α but the latter does not have an endocrine effect (26, 27). In contrast, the TNF-α produced by mesenteric AT contributes to inflammation in the mesentery (28). Most recently, AT has also been identified as a site of miRNA production; miRNAs with endocrine activity are packaged into exosomes and then released into the circulation (11).

#### Metabolic and Immune Cells Coexist in AT

The influence of AT on acute or chronic immune responses depends on the resident immune cells. As mentioned above, AT contains differentiated adipocytes and stromal/vascular cells. Virtually all the known types of immune cell have been detected in WAT: eosinophils, neutrophils, mast cells, macrophages, T lymphocytes (including regulatory T cells, CD4 and CD8 αβ T cells, γδ T cells, and natural killer (NK) T cells), type I and II innate lymphoid cells, and invariant NK cells (29). Remarkably, the presence of immune cells is required for the full metabolic activity of AT under normal conditions. Macrophages (including M2 anti-inflammatory and M1 pro-inflammatory subsets) and T lymphocytes are the most numerous immune cells in AT. The macrophages clear dead adipocytes, buffer lipids and contribute to angiogenesis (30). The regulatory CD4 T cell (Tregs) fraction is highly represented in lean AT in some mouse models (31). Other Th2-like cells (e.g. eosinophils) also help to limit excessive inflammation and modulate lipogenesis (32). Importantly, a degree of inflammation in AT is essential for homeostasis (2). Whereas adipose stem cells (ASCs) are crucial for metabolic homeostasis, they also send immunomodulatory signals to immune cells. In an *in vitro* study of the immunomodulatory effect of ASCs on macrophages isolated from osteoarthritic synovial tissue, the secretion of prostaglandin E2 favored the M1-to-M2 transition (33). *In vitro* culture of human ASCs with T lymphocytes also induces immunosuppression through the PD1/PDL1 Gal-9/TIM-3 pathways (34). The interplay between immune and metabolic cells is subtle, and metabolic stress induce changes in the immune cells’ composition and functions.

In addition to having key roles in metabolic regulation, these immune cells notably exhibit immune and cytokine-secreting activities that might contribute directly to systemic inflammation and/or sustain/exacerbate local inflammation in AT. Recent research has suggested that tissue-resident T lymphocytes contribute to secondary immune responses by migrating towards lymphoid sites, although the underlying mechanisms have not been fully evaluated (35, 36). If this process occurs in AT (e.g. with the release of memory T cells into draining LNs), it might constitute a novel way in which AT contributes to chronic immune activation.

#### Functional Differences Between SCAT and VAT

Although SCAT and VAT are morphologically similar; the latter is more densely vascularized and innervated and contains larger numbers of adipocytes, and immune cells (37).

##### Metabolic Differences

A growing body of evidence suggests that SCAT and VAT differ in their metabolic characteristics (38, 39) and thus their functions. VAT has a lower proportion of preadipocytes and a higher proportion of large adipocytes (39). Furthermore, VAT adipocytes are more insulin-resistant than SCAT adipocytes but also have a higher metabolic rate. In fact, VAT is more sensitive to adrenergic stimulation and thus more prone to lipolysis and FFA release. In contrast, SCAT buffers levels of FFAs and triglycerides. The progenitor cells’ adipogenic capacity is closely linked to metabolic changes. In obesity, hypertrophic adipocytes (which contribute to contribute to insulin resistance) are frequently observed in VAT (40), and VAT expansion is correlated with the onset of metabolic disorders (41, 42). Adipocyte precursors isolated from SCAT displayed a higher adipogenic potential than those from VAT (40). In rodent models of obesity, it has been shown that SCAT is metabolically beneficial. Indeed, the intra-abdominal transplantation of SCAT ameliorates several metabolic parameters, including insulin resistance (43). Likewise, removal of VAT can prevent the onset of insulin resistance (44).

##### Endocrine and Immune Differences

VAT *vs.* SCAT differences in endocrine activity have been documented in both healthy lean individuals and obese individuals (**Table 1**). The differences are more pronounced in the context of obesity. In healthy individuals, differences in C-C chemokine receptor type 2 (CCR2) and macrophage migration inhibitory factor (MIF) (45, 46) have been reported, and adiponectin is secreted more by VAT than by SCAT. However, VAT is prone to being pro-inflammatory in response to obesity. IL-6 is secreted more by VAT than by abdominal SCAT, whereas leptin is secreted more by SCAT. On the cellular level, no difference in the number of macrophages has been detected, although VAT has a more pro-inflammatory profile than SCAT (47). In mice fed a high-fat diet, macrophage infiltration is increased rapidly in VAT but not in SCAT (48, 49). It is noteworthy that macrophages and inflammation also contribute to the remodeling of the extracellular matrix (ECM). In VAT in obese individuals, increased fibrosis (characterized by the excessive production of ECM components) interrupts normal metabolic functions and accentuates inflammatory responses (48, 49) (see the following section).

#### Differences Between Subcutaneous Upper Versus Lower Fat

The SCAT is the greater fat mass in healthy individuals, and differential capacity for certain subcutaneous regions are described, notably lower-body (gluteal fat, subcutaneous leg fat, and intramuscular fat), and upper-body subcutaneous fat (including abdominal SCAT) (50). Although the accumulation of upper fat (abdominal obesity, including visceral and abdominal subcutaneous) is associated with the development of cardiovascular disease and type 2 diabetes mellitus, lower-body fat has protective properties that are associated with an improved cardiometabolic risk profile in men and women (51). However, the underlying mechanisms for the functional differences between upper and lower-body AT remain elusive (52). The observation that regional characteristics are retained *in vitro* strongly indicates that the differences in adipocyte functions are inherent to the depot rather than a consequence of the local microenvironment. Abdominal and gluteofemoral SCAT present differentially-expressed developmental genes (53, 54) including members of the homeobox (HOX) family, HOX-domain encoding genes and T-box genes. In addition, lower-body AT is characterized by low lipid mobilization (55, 56) through α-adrenergic (antilipolytic) receptors. The release rates of non-esterified fatty acid (NEFA) are lower, in accordance with the reduced overall turnover of the triglyceride pool of this tissue (52). On the other side, abdominal SCAT is characterized by smaller adipocytes, higher expression of adipogenic, lipolytic, and mitochondrial genes associated with lower oxygen consumption (57) and the capacity to recruit new adipocytes seems limited in this tissue. Thus, different metabolic pathways regulate lipid metabolism in upper- *versus* lower-body fat and these tissues respond differently to weight gain in favor of a causality between lower-body fat accumulation and reduced risk of cardiometabolic diseases.

### The Plasticity of AT and Fibrosis in the Context of Obesity/Metabolic Disorders

AT is highly plastic, so that it can regulate energy influx and efflux. Adipocytes react to energy overload by initiating hypertrophy (an increase in cell size, due to lipogenesis and triglyceride accumulation) and hyperplasia (an increase in cell number, due to adipogenesis); both of these processes are associated with metabolic and endocrine changes, as extensively reviewed elsewhere (13, 58, 59). During AT expansion, the ECM requires remodeling to accommodate adipocyte growth. In AT, the ECM is characterized by collagens; these are produced mainly by adipocytes but also by stromal/vascular cells. Adipocytes are maintained within an ECM network whose deposition is impaired during obesity. Fibrosis can mechanically limit tissue plasticity and contribute to metabolic impairments (60). In obese individuals, the presence of pericellular fibrosis and fibrosis bundles was negatively correlated with hypertrophy (61). In this context, fibrosis is associated with inflammation and insulin resistance (60, 62, 63). Moreover, ECM deposition was found to be higher in SCAT than in VAT in both lean and obese subjects (63),, although adipocyte hypertrophy in VAT coincides with increased adipocyte death, formation of crown-like structure, inflammation, and insulin resistance (60, 64, 65). Simultaneous changes in the immune cell compartment are also observed and drastically modify the AT’s secretory profile (66). Although adipocytes secrete some pro-inflammatory cytokines (IL-6, TNFα, and IL-8) at low levels, immune cells account for most of the cytokine secretion.

#### Immune System Activation and Inflammatory Activity of AT in Obese Contexts

AT inflammation is a standard feature of obesity, and mainly involves AT macrophages. Macrophage accumulation and a progressive shift from an M2 phenotype to an M1 phenotype are observed in AT during obesity (49). This translates into a shift from the secretion of immunosuppressive cytokines [such as IL-10 and transforming growth factor β (TGF-β)] to the secretion of pro-inflammatory cytokines (such as IL-6, TNF-α, MCP-1, IL-12, IL-23, and IL-1β). However, several mechanisms are involved in the shift in the AT’s inflammatory profile; they include the direct impact of adipocyte dysfunction and M1 differentiation (both through the adipocyte’s cytokine production (67) and lipid buffering) and the local influence of other immune events, such as neutrophil accumulation, Th1 bias (leading to greater IFN-γ production), and a reduction in the proportion of Tregs. The recruitment of neutrophils (rather than eosinophils) also contributes to the AT’s pro-inflammatory secretion profile; the production of superoxide, elastase and myeloperoxidase by neutrophils and IL-1β production contribute to the inflammatory response (68). The Th2-Th1 shift and the recruitment of CD8 T cells increase the level of IFN-γ production, favors M1 macrophage polarization, and activates adipocytes (69). Additionally, Th17 cells are also recruited in AT in obese contexts, under the combined influences of AT macrophages, ASC and adipocytes (70). Recruitment of NK cells is also described in visceral (epididymal) but not subcutaneous AT in the HFD mouse model. The upregulation of NK activating ligands by adipocytes contributes to the increase in NK cells in SCAT, that will subsequently contribute to M1 macrophage differentiation (48, 71). The exact sequence of events is still not fully known, although neutrophils and CD8 T cells appear to have crucial roles in the initiation of local inflammatory responses (68, 72) because they are detected before the accumulation of macrophages. Moreover, inflammatory macrophages disrupt ECM homeostasis, which leads to fibrosis (2). Lastly, advanced glycation end-products (AGE) that are increased in obese diabetic patients, impact both adipocyte and immune cells functions, as both cell types express the receptor for AGE (RAGE) (73, 74). On the whole, inflammation in AT is triggered by several integrated mechanisms and involves the various cell subsets in the AT, i.e. immune cells, adipocytes, and (presumably) ASCs - a shift towards a more inflammatory profile has not been described for the latter. In a vicious circle, the concomitant pro-inflammatory signals produced by these various cell types help to drive local inflammation.

#### Contribution of AT to Low-Grade Inflammation During Obesity

Obesity is associated with low-grade inflammation (75–77) and insulin resistance (78). It is generally acknowledged that the secretion of adipokines by AT contributes to overall inflammation, although the exact contribution of a given pro-inflammatory cytokine to local and/or systemic inflammation is difficult to evaluate. The complexity of the inflammatory network that develops in AT (involving intricate, redundant, time-framed interactions between pro-inflammatory signals) is an initial hurdle. The integrated metabolic and inflammatory processes that drive each other in the AT (79) and thus amplify local inflammation constitute a second hurdle. Lastly, a third level of complexity relates to the opposing influences of cytokines produced by metabolic vs. immune cells, since the results appear to depend on the type of producing cell for IL-6 (80) and type I interferon [as reviewed in (81)] (82, 83). Although the secretion of adipokines by AT contributes to overall inflammation, the low-grade inflammation observed in the blood is due to many inflammatory factors, including lipotoxicity, disruption of gut microbiota, and systemic immune activation. The severity of obesity and the development of metabolic comorbidities (84) must also be taken into account.

## Causes of Adipose Tissue Alterations During HIV Infection

HIV infection in ART-naive patients is characterized by wasting, affecting both muscle and fat mass. ART-naive patients have lower SCAT volume and decreased whole tissue expression of pro-adipogenic genes compared to HIV-negative controls together with decreased mitochondrial DNA (mtDNA) content and reduced expression of mitochondria-encoded proteins that are required for adequate adipocyte metabolic function (85). Different AT alterations were observed in HIV-infected patients receiving ART. Although HIV impacts AT biology per se and metabolic profile (86), these changes were mainly attributed to the ART. The first-generation drugs taken by patients had lipotoxic effects; the resulting lipoatrophy gave rise to a syndrome called “ART-related lipodystrophy”. Once lipotoxic molecules had been replaced by less toxic drugs, patients progressively gained fat (mainly on the trunk). The broad use of the newer class of integrase strand transfer inhibitors (INSTIs) now means that some patients can increase their overall fat mass. Understanding why AT mass, distribution, and function are often altered in HIV-infected patients (regardless of the type of ART) is a real challenge. Viral persistence certainly has an important role, together with HIV-related changes in the immune context inside and outside AT. The fact that the patients’ fat alterations became severe only after the introduction of ART molecules (i) emphasizes the drugs’ essential contribution to harmful changes and (ii) suggests that HIV and some antiretrovirals (ARVs) act in synergy on adipocytes and other adipose cells to induce metabolic and inflammatory dysfunctions in AT and thus trigger adverse events in other tissues.

### HIV-Related Fat Alterations: The Impact of HIV Infection on AT Biology

**Figure 1:**
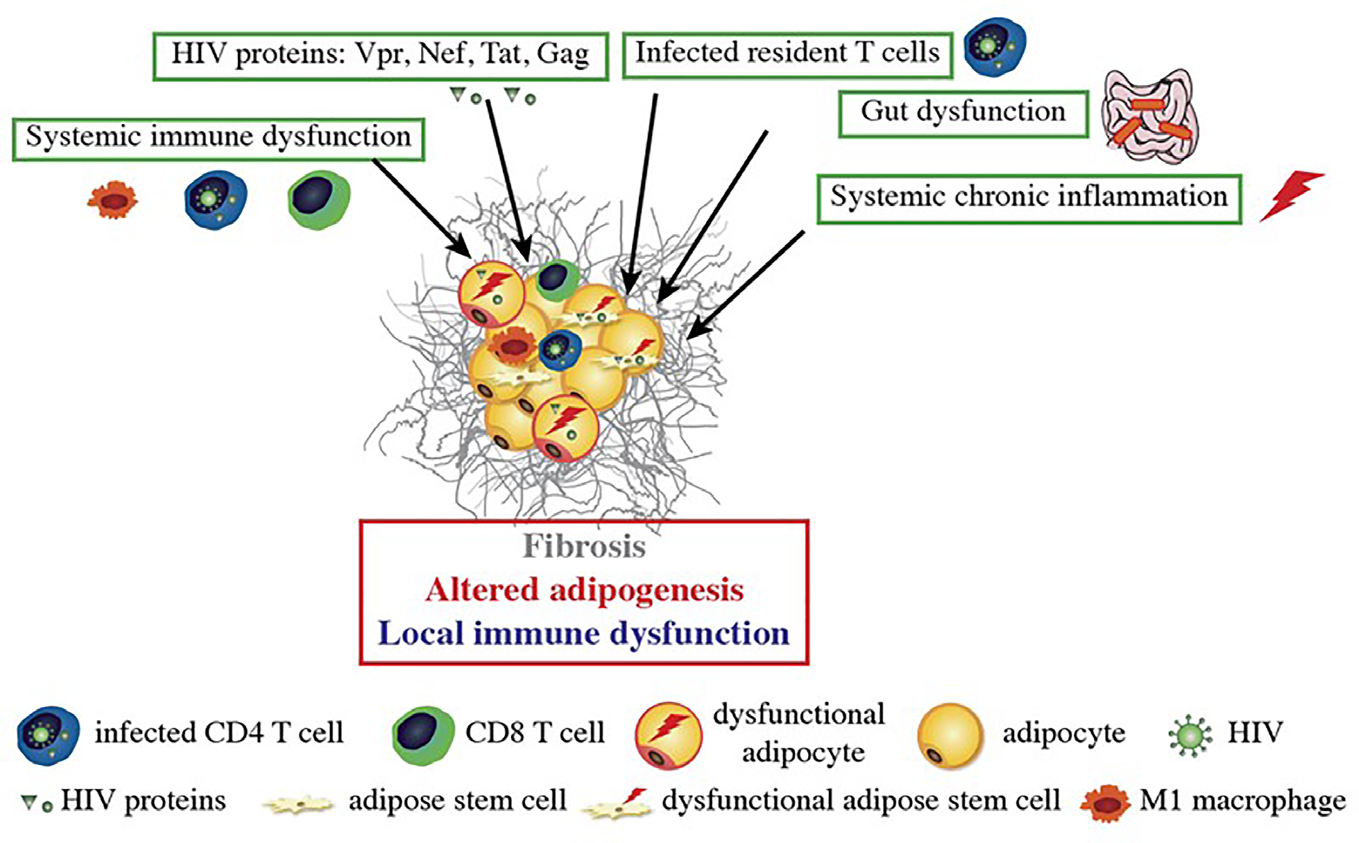
HIV-related alterations in AT. HIV itself and the viral proteins produced by infected cells induce fibrosis and alter adipogenesis and local immune functions. HIV proteins are secreted by infected cells within AT and can impact the nearby adipose stem cells (ASCs) and adipocytes. Dysfunctional ASCs present mitochondrial dysfunction, elevated levels of oxidative stress and a profibrotic phenotype. The proteins impede the ASCs’ ability to differentiate into adipocytes; this leads to dysfunctional adipocytes with low expression of adipogenic markers, lipid accumulation, and leptin and adiponectin secretion. HIV-protein-treated adipocytes also acquired a pro-inflammatory phenotype that is involved in local inflammation and immune dysfunction. Systemic factors (gut dysfunction, systemic chronic inflammation, and immune dysfunction) also contribute to AT dysfunction, with the induction of CD8 T cells, macrophages infiltration, and a shift towards a pro-inflammatory M1 macrophage profile.

Although ART is clearly associated with adverse events, the presence of metabolic alterations in treatment-naive, HIV-infected people (86) suggests that the virus itself also has an impact on AT biology. All the studies performed in the 2000s found that adipocytes were not infected by HIV (87, 88) and that no HIV components were present in these cells. However, other HIV-related mechanisms have been identified (**Figure 1**).

#### Viral Proteins

Since viral proteins are present in patients with no detectable viral load (87, 89, 90), it has been suggested that Vpr, Nef, Tat and Gag have an impact on AT.

The mechanisms whereby viral proteins might affect adipocyte functions have been investigated *in vitro*. Nef, the accessory protein Vpr, and the regulatory protein Tat have been shown to alter adipogenesis (85, 91, 92) and might also contribute to the onset of insulin resistance in adipocytes (92). Interestingly, some studies have shown that Tat induces mitochondrial dysfunction [probably through mitochondrial membrane permeabilization and the generation of mitochondrial reactive oxygen species (ROS) (93)], which might account for the observed AT dysfunction and insulin resistance. It was established that HIV infection upregulated the expression of collagen 6, fibronectin and the profibrotic factor TGF-β in SCAT and (to a lesser extent) VAT. These observations were confirmed *in vitro*, where Tat and Nef promoted the acquisition of a profibrotic phenotype and increased the production of ECM components by adipocytes and their precursors (91).

The impact of the viral proteins on immune cells is well documented in general but has not been specifically investigated for AT-resident immune cells. It is known that the intracellular diffusion of viral proteins influences the function of T cells (94–96) and macrophages (97, 98). Nef binds to CXCR4 and modulates T cell activation (99). Lastly, T cells are activated (as expected) after exposure to viral proteins but the impact on the immune response in AT has not been extensively investigated.

#### Direct Infection of AT-Resident CD4 T Cells

The observation of high numbers of immune cells (and notably CD4 T cells and macrophages) in AT in obese individuals prompted reexamination of whether AT was directly infected by HIV. Couturier et al. detected HIV in the stromal vascular fraction prepared from AT (100). Subsequently, HIV (and SIV, in macaque models) were detected in the CD4 T cell fraction (101, 102) in both viremic and controlled ART-experienced patients. Lastly, several research groups have confirmed that the virus found in AT is replication-competent (102, 103). It is noteworthy that unambiguous data on the macrophage fraction are not available: the virus was found in the viremic macaque model but not in samples from ART-experienced, HIV-infected patients (102).

#### Systemic effects of HIV infection on AT functions

Although HIV is undetectable in the blood of ART responders, some dysfunctions persist. An ART-controlled HIV infection is known to be associated with low-grade chronic inflammation and persistent immune dysfunction (104–107), although the extent of these defects is subject to debate. Schematically, three main HIV-related factors may indirectly impact AT functions: gut dysfunction, immune dysfunction, and inflammation.

##### Gut Dysfunction

A crucial event during the first stage of an HIV infection is the severe, rapid depletion of the CD4 T cell compartment in the gut. ART enables only partial restoration of the gut mucosa (108) and does lasting damage to the gut’s functional properties. In some ART-controlled patients, gut dysfunction impacts both the immune responses and the AT functions. The intactness of the epithelial barrier is weakened, leading to greater microbial load and viral persistence that are factors in chronic immune activation and inflammation. The disruption of the gut microbiota (109) modulates AT functions and subsequently impacts AT immune cells. Indeed, gut microbiota has a causal role in the development of obesity and associated metabolic disorders (12, 110, 111): microbiota transplanted from obese mice induces obesity. Given the central role of gut microbiota on metabolic and immune responses, multiples pathways are presumably involved (increase in LPS that may directly favor AT expansion, changes in metabolites and miRNA production by the gut, resulting in changes in AT immune cells inflammatory potential and in metabolites, adipokines and miRNA production by AT…). We currently lack data on changes in metabolite and miRNA production in the gut during chronic ART-controlled HIV infection.

##### Immune Dysfunction

The immune responses that develop during HIV infection lead to a progressive loss of cell function. For example, HIV-specific CD8 T cells exhibit an exhausted profile (112). The loss of immune function has several known causes, and others probably remain to be found. The following causes are commonly cited: (i) persistent antigenic stimulation; (ii) dysregulated immunoregulation (the important immunosuppressive population of Tregs are targeted by HIV, albeit to a lesser extent than conventional CD4 T cells, and thus control the immune responses less tightly; (iii) immune activation caused by exposure to a pro-inflammatory environment, (iv) the differentiation of T cells into less functional cells (such as senescent and/or exhausted cells), which in turn favors antigen persistence. Given that the AT is also a site of immune cell accumulation (predominantly CD4 T cells and immunoregulatory T cells in lean individuals), immune dysfunction during chronic HIV infection can particularly disturb the AT-resident immune cells’ immune and metabolic functions.

##### Chronic Inflammation

**Figure 2:**
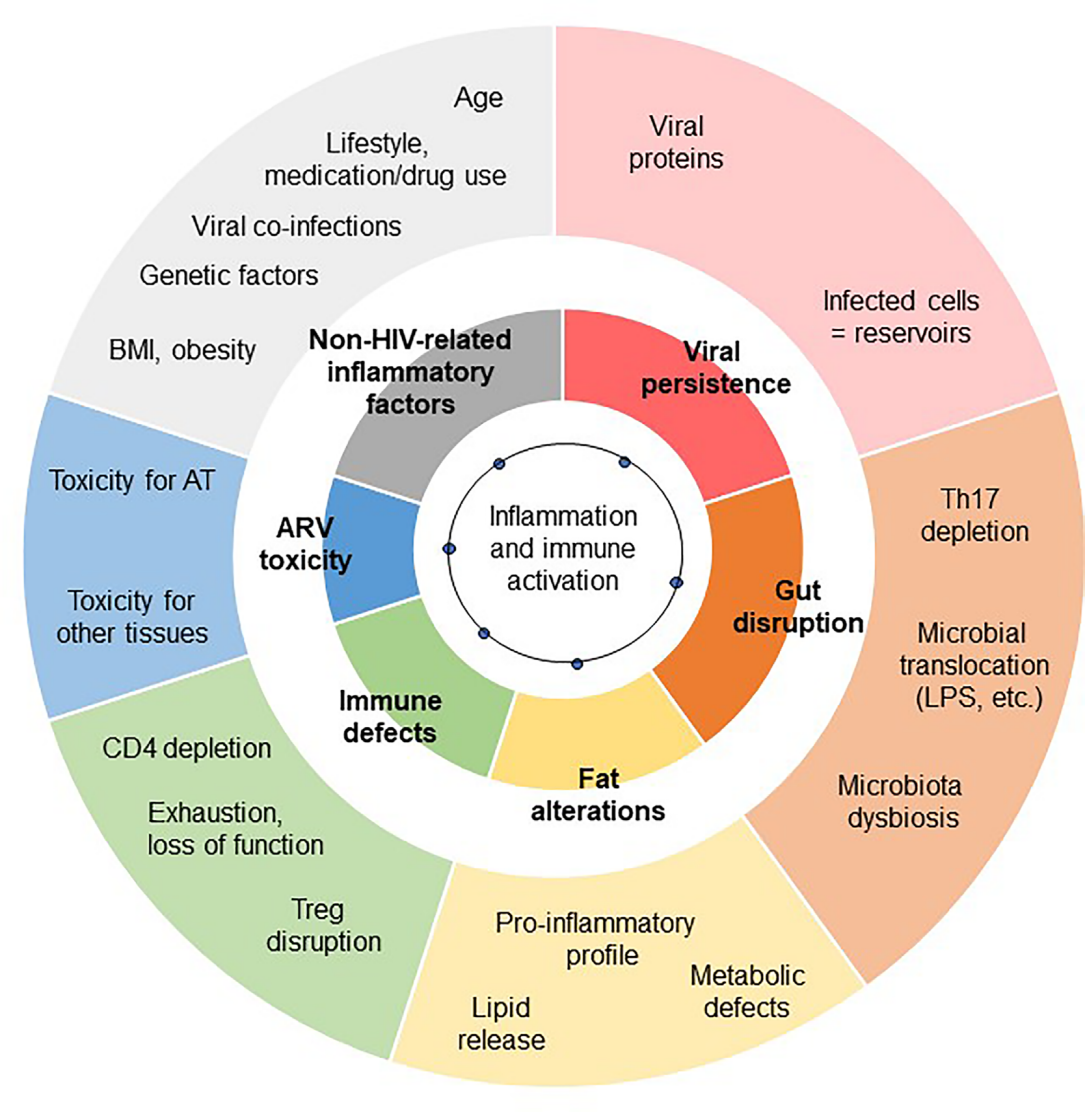
Contribution of AT dysfunction to HIV-related low-grade inflammation. The exact degree of low-grade inflammation associated with chronic, controlled HIV infections remains subject to debate. The inflammation profile depends on the biomarkers studied and the clinical context of chronic HIV infection (including the time of ART initiation, and the severity of the primary phase of the infection). However, various triggers of inflammation are commonly reported to contribute to low-grade chronic inflammation: viral persistence, gut disruption, immunoregulatory defects, and non-HIV related comorbidities. Each of these main factors (shown in the inner circle) has several subfactors (shown in the outer circle). Fat alterations and the toxicity of ARVs also contribute.

The low-grade inflammation observed in chronic HIV-controlled patients has several causes. It combines immune dysfunctions (CD4 depletion, which is partially restored; defects in immunomodulatory mechanisms, and gut disruption), metabolic inflammation (“meta-inflammation”), inflammation associated with pathogen persistence/overload (leading to constant immune activation, i.e. “infectious inflammation”) and “inflammageing” (HIV infection has been linked to accentuated ageing) (3). The residual inflammatory signature also depends on several factors, including the CD4 T cell count (both at the nadir and after treatment), the timing of ART, the patient’s age and gender of the patients, and non-HIV-related factors (overweight, viral co-infections, smoking, alcohol consumption, recreational drug abuse, drug toxicity, etc.) (106, 113). Regardless of the exact nature of the inflammation that develops during a chronic ART-controlled HIV infection, the AT is highly sensitive to the inflammatory environment. As described above, systemic inflammation may contribute to or initiate local inflammation in AT. Reciprocally, AT inflammation may contribute to the systemic low-grade inflammation observed in HIV-infected patients (**Figure 2**). Importantly, the amplitude and nature of this chronic inflammation is also highly dependent on effective ART. This therapy restores the CD4 T cell count fully or partially (as defined by the patient’s status as an “immune responder” or an “immune nonresponder”) and reduces microbial translocation, viral persistence in reservoirs, and inflammation. Although ART is highly beneficial, it is also associated with metabolic side effects – notably within AT. ART can thus drastically change the nature of the inflammation that persists during chronic HIV infection.

### ART-Related Lipodystrophy

#### Description

Prior to the development of ART, HIV-infected patients were seen to experience severe changes in body composition (including loss of muscle and AT) during the first few years of HIV infection. This resulted in cachexia, especially when patients had progressed to AIDS (85).

The introduction of the first thymidine nucleoside reverse transcriptase inhibitors (NRTIs), such as zidovudine and stavudine, enabled partial control of the HIV infection and delayed the onset of AIDS. Next, the introduction of protease inhibitors (PIs) enabled the “resurrection” of patients with AIDS who were already in a state of profound, life-threatening immunodeficiency. Triple therapy (a combination of two NRTIs and a PI) enabled real control of the virus, with an increase in the CD4 cell count and a marked decreased in the HIV load. However, patients with controlled infections commonly lost fat, even though the muscle mass was unaffected. This lipoatrophy was observed at all SCAT locations (the limbs, trunk, face, and buttocks). In particular, facial lipoatrophy gave the patient a striking, stigmatizing, cadaveric appearance – a hallmark of HIV infection. Furthermore, the levels of visceral fat variously decreased or increased, giving a mixed lipodystrophy phenotype (peripheral lipoatrophy plus central lipohypertrophy). Lastly, some patients developed a “buffalo neck”, with the accumulation of fat at the back of the neck.

It was soon determined that thymidine NRTIs (mainly stavudine but also zidovudine) were responsible for the lipodystrophy, and these drugs were replaced by NRTIs with little or no AT toxicity. However, the features of lipoatrophy can persist for years (with long-term cardiometabolic consequences) after a patient has switched from thymidine NRTIs.

#### The Impact of First-Generation ARVs on Adipocytes and Their Progenitors

**Figure 3:**
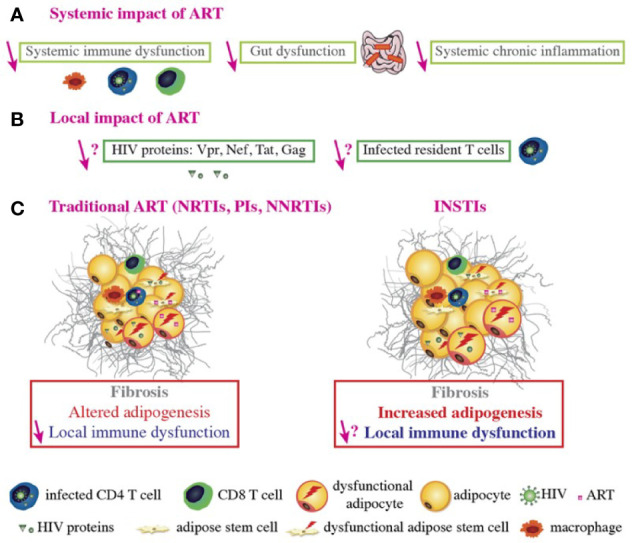
ART-related fat alterations. ART controls HIV replication, improves systemic immune function, decreases chronic inflammation, promotes immune reconstitution and function, improves gut function and integrity, and decreases the viral load and viral protein concentration in the blood **(A)**. However, it is not known whether ART decreases the number of infected T cells or the viral protein concentration within AT **(B)**. Regarding conventional ART, the NRTIs stavudine and zidovudine, some PIs boosted by ritonavir, and the NNRTI efavirenz (EFV) induce alterations in AT such as fibrosis, altered adipogenesis, and local immune dysfunction. ART does improve immune function and expression levels of some adipogenic markers (e.g. PPARγ) in the AT, relative to treatment-naive HIV-infected patients. However, adipogenic marker expression is still lower than in non-infected people. ART induces mitochondria dysfunction and alters adipogenesis in both ASCs and adipocytes. ART-treated adipocytes present with oxidative stress and insulin resistance. Furthermore, some ARVs induce pro-inflammatory adipokine/cytokine secretion by adipocytes, ASCs and macrophages and CD8 T cells accumulation; this contributes to AT immune dysfunction and inflammation. It was recently reported that some INSTIs can be associated with fat and weight gain, adipogenesis, fibrosis, and adipocyte hypertrophy/dysfunction, with increased insulin resistance *in vitro*. INSTIs also induce collagen production, mitochondrial dysfunction, and oxidative stress in both ASCs and adipocytes, which might promote fibrosis. Nonetheless, the local impact of INSTIs on immune cells within the AT remains to be determined **(C)**.

The mechanisms underlying the effects of individual drugs on AT have been investigated *ex vivo* and *in vitro*. The *ex vivo* studies of fat samples from patients were limited by the fact that none of the latter were being treated with a single ART and that the triple therapy at that time constituted of a PI and two NRTIs. In general, only subcutaneous fat samples were available. The *in vitro* studies of each ART on adipose cells in cultured models were limited by the absence of a true, three-dimensional AT milieu comprising the different cell types (**Figure 3**).

##### The First-Generation Thymidine NRTIs Stavudine and Zidovudine

*Ex vivo* analyses of samples taken from lipoatrophic areas indicated that AT was deeply affected, with small adipocytes, fibrosis, and crown-like structures indicating the infiltration of macrophages around dead adipocytes (114, 115). This situation was unusual because crown-like structures are observed during obesity, when adipocytes are enlarged. In cases of lipoatrophy, these histological features pointed to a toxic effect of ART on adipocytes. Furthermore, expression levels of the main adipogenic factors (peroxisome proliferator activated receptor γ (PPAR-γ), CCAAT/enhancer-binding protein alpha (C/EBP-α), and sterol regulatory element-binding transcription factor 1 (SREBP-1)), adipose markers, adiponectin, and leptin were abnormally low. Greater levels of inflammation were characterized by increased gene and protein expression of proinflammatory cytokines (like IL-6 and TNF-α) by stressed adipocytes and immune cells infiltrating the AT. Furthermore, the AT from lipoatrophic subjects has a low mitochondrial DNA (mtDNA) content and low expression levels of the mitochondria-encoded proteins required for adequate metabolic function in adipocytes (116, 117). In end-stage lipoatrophy, all the subcutaneous ASCs and adipocytes were destroyed. In contrast to SCAT, few studies have evaluated alterations in VAT in ART-treated patients. However, NRTIs appear to be less toxic for VAT adipocytes than for SCAT adipocytes (85).

The role of thymidine NRTIs was further investigated in patients who switched from these drugs. Progressive AT recovery was observed, although recovery from severe lipoatrophy was often only partial - probably due to irreversible exhaustion of the ASCs in subcutaneous fat (118).

##### The First-Generation PIs Indinavir and Nelfinavir

Studying PIs in clinical AT samples was difficult since the patients were receiving two NRTIs (including one thymidine NRTI) at the same time as a PI. Therefore, most of the information on the effects of indinavir and nelfinavir has been obtained *in vitro* (see below). It has been suggested that some PIs have a role in the shift from a brown/beige phenotype to a white phenotype because the expression of the micro-RNA processing enzyme Dicer was found to be low in lipoatrophic areas, and this decrease was proportional to the duration of PI use.

#### ART-Related Changes in Fat With Combinations of Today’s ARVs

##### Description

More recently, HIV-infected patients with long-term control of the infection were generally receiving triple therapy with two NRTIs and a PI or a nonnucleoside reverse transcriptase inhibitor (NNRTI, such as efavirenz). As the treated patients aged, however, the prevalence of comorbidities increased. The patients often gained weight and fat, with the classical truncal distribution seen during ageing (85, 119). These changes were associated with adverse cardiometabolic outcomes, as atherosclerotic cardiovascular disease (CVD), diabetes, and non-alcoholic fatty liver disease (NAFLD).

This increase in trunk fat is seen in both women and men and in all ethnic groups. It can result in obesity, particularly when the initial bodyweight is already high (as observed frequently in North America and less so in Europe). As the person ages, the fat gain is often accompanied by the loss of muscle mass. This situation is called sarcopenic obesity and is associated with harmful cardiometabolic outcomes.

Nowadays, the recently developed class of INSTIs is widely used to control HIV infections. Although the INSTIs are effective and safe, combination therapy (with the addition of an NRTI) is typically required to control the virus. However, some patients gain weight and fat mass (mainly on the limbs, trunk, and abdomen) and become obese (85).

Weight gain can affect treatment-naive patients initiating ART. A large proportion of this gain is due to a return to good health; patients with a low CD4 and a high viral load gain the most weight during the first two years of ART. Nevertheless, this weight/fat mass gain is excessive in some individuals taking an INSTI [mainly dolutegravir (DTG) and bictegravir], especially in women (vs. men) and African ethnic groups (vs. Caucasians) (120, 121). Weight gain is also observed in some ART-controlled patients who switch to an INSTI; female sex and older age are risk factors for this increase. Nevertheless, it is unclear why some patients (but not others) gain weight disproportionally. Recent studies have indicated that along with INSTIs, commonly used “older” drugs can also modulate weight gain. For example, the NNRTI efavirenz and the NRTI tenofovir diproxil fumarate (TDF) have been linked to lower weight gains, while tenofovir alafenamide (TAF, a derivative of TDF that concentrates within cells) is associated with a greater weight gain (121).

##### The Impact of INSTIs and TAF/TDF on Fat

In AT, adipocytes can sequester ARTs inside the adipocyte lipid droplet, which contributes to viral persistence (87). Adipocytes have consistently been shown to reduce the antiviral efficacy of TDF and TAF *in vitro*, and some INSTIs have been found in AT (122).

###### Effect of INSTIs

Recent *ex vivo* studies have found that fibrosis, adipocyte size, and adipogenic marker expression are greater in SCAT and VAT samples from INSTI-treated macaques than in samples from untreated animals. Similarly, SCAT and VAT from obese INSTI-treated HIV-infected patients show higher levels of fibrosis than samples from INSTI-naïve obese patients (85, 123).

DTG has been detected within adipocytes and in the stromal vascular fraction prepared from VAT (122), suggesting that it has access to ASCs and adipocytes *in vivo*. Raltegravir (RAL) has a high level of tissue penetration and is therefore likely to accumulate in AT (124). These features might explain the fat mass gain in treated patients (**Figure 3**).

Recent results suggest that INSTIs can decrease SCAT inflammation. In the OBEVIH cohort of obese subjects undergoing bariatric surgery, the level of inflammation was lower in HIV-infected patients treated with an INSTI than in patients not receiving an INSTI and in HIV-negative controls (Pourchez V, unpublished data). Accordingly, our study of SCAT samples from HIV-infected patients switched from a PI-containing regimen to combination therapy with RAL and the CCR5 inhibitor maraviroc revealed very low expression levels of genes involved in T lymphocyte signaling (125).

###### Effects of TAF vs. TDF

Differences in pharmaceutical formulation mean that TAF has lower circulating concentrations but higher intracellular concentrations than TDF. It remains to be determined why TDF prevents weight gain and TAF enhances it. However, it is possible that these effects are related to differences in the level of drug-induced oxidative stress.

### A Third Factor: The Host Status Prior Infection

#### Inflammation Is an Integrative Process

It is challenging to distinguish between the respective effects of HIV infection, ART toxicity and personal factors on the inflammatory profile developing in ART-controlled HIV-infected patients. The interplay between HIV and the ARVs are multiple. Firstly, ART reduces viral persistence and virus-related immune defects, suggesting that the persisting low-grade inflammation observed in ART-controlled patients may partly relate to ARV-toxicities when immune restoration is achieved. Among host-related factors, the severity of the primary phase, the size of the pre-established viral reservoir may affect the quality of the immune restoration and also participate to the development of low-grade inflammation. Immune and tissue-specific natural senescence together with the presence of age-related comorbidities may also contribute to the development of an exacerbated immune responses. In aged patients, ART toxicity is increased probably due to reduced capacities to detoxify and eliminate drugs (kidney and liver failures) and to the prescription of other drugs (polypharmacy).

Importantly, patients with efficient immune restoration present inflammatory profile close to healthy individuals (106, 113), suggesting a limited impact of currently used ARVs per se on inflammation. However, the question of the relative impact of HIV infection and ARV remains open when considering patients with partial immune restoration. One may hypothesize sequential impacts of HIV infection and ARV on AT. It could be hypothesized that ARV may impact differently AT that was previously disrupted by HIV infection compared to uninfected AT, possibly acting in synergy to amplify adipose dysfunction. The same notion may apply to other infections that target AT (tuberculosis, CMV, …) and may alter the metabolic and immune parameters of AT. Lastly, because HIV persist in AT even after ART induction, one may consider the combined impacts of HIV infection and ARVs on the alteration of AT. These questions are difficult to address *ex vivo* since ARV are prescribed only to HIV-infected patients and very limited data are available in animal models regarding the study of ARV toxicity with/out HIV/SIV infection, and the associated inflammation.

#### An Important Factor: The Host Metabolic Status Prior Infection

Importantly, the impact of HIV infection and ARV on AT may also directly rely on the host metabolic status prior infection. In that respect, preexisting obesity may exacerbate the pathophysiology of HIV infection and the toxicity of ARVs. Studies in NHP demonstrate the higher severity of SIV pathogenesis in animals fed with high fat diet with resulting cardiovascular (CV) and liver alterations (126, 127), but ARV were not administered in this setting. Data in humans are obviously less consensual. Before ART’s availability, HIV infected patients with higher BMI showed slower HIV progression compared to underweight patients (128, 129), presumably due to delayed wasting/cachexia effects. In ART-treated patients, obese or underweight patients exhibited higher cumulative mortality rates, delineating an optimal BMI range between 24 and 28 (130) and obesity has been identified as a risk factor for multimorbidity (131). Regarding the impact of obesity on CD4 T cell reconstitution, patients with higher BMI before ART initiation exhibit higher (132, 133) or comparable (134) immune reconstitution. This apparent discrepancy between mortality rate and CD4 T cell restoration exemplified the diverse impacts of obesity which is associated simultaneously with positive and negative effects. However, the impact of obesity on the toxicity of ARV remains ill-documented. It has been hypothesized that the sequestration and metabolism of ARV may differ in obese versus lean patients. ART plasma concentrations were lower in obese patients, although not impacting the viral control (135).

As a whole, the close interplay between HIV infection, ARV toxicity and metabolic status prior infection and/or following ART introduction may be considered as a combination rather than as independent factors of inflammation.

## Phenotypic and Mechanistic Changes in AT

### Metabolic Changes

#### Local Effects

A number of *in vitro* studies have sought to describe the mechanisms involved in the detrimental effects of individual PIs, NRTIs and NNRTIs on adipogenesis, insulin signaling, adipokine secretion, and apoptosis (85).

*In vitro* studies of adipocytes cultured with individual thymidine NRTIs showed that stavudine and (to a lesser extent) zidovudine were toxic for AT. The drugs inhibited the mitochondrial DNA polymerase (required for mtDNA replication) and also induced severe oxidative stress. Thus, toxic effects on mitochondria might result in an energy deficiency and high levels of oxidative stress (136). Inter-individual differences in sensitivity to the lipoatrophic effect of stavudine or zidovudine might be related (at least in part) to mtDNA haplogroups, some of which are variously associated with a greater risk of lipoatrophy (the H, I and K haplogroups) or a lower risk (the T and W haplogroups) (137, 138).

The effects of first-generation PIs on adipose cells have also been evaluated *in vitro*. These effects might result (at least in part) from the inhibition of the enzyme ZMPSTE24, which is responsible for the maturation of the precursor prelamin A into the nuclear protein lamin A. First-generation PIs were able to inhibit the enzyme, which led to the accumulation of prelamin A around the nucleus and to mislocalization of SREBP-1 (139, 140). The accumulation of prelamin A favored cell senescence, adipocyte dysfunction, and insulin resistance. First-generation PIs could also induce endoplasmic reticulum (ER) stress. Furthermore, a recent study showed that both short‐ and long-term treatment with the first-generation PI lopinavir promoted whitening of brown adipocytes differentiated *in vitro*. This shift impeded fatty acid oxidation and favored insulin resistance (141–143).

Efavirenz has been used for a long time and is still given to HIV-infected people today. However, it can reduce adipogenesis and adiponectin expression whilst increasing the expression of proinflammatory markers (144).

More recently, several research groups have started to identify and characterize the mechanisms involved in the INSTIs’ effect on weight/fat gain. The etiology of INSTI-related weight/fat gain is unclear but may include indirect effects on thermogenesis, appetite or energy regulation, or a direct effect on AT. We have observed that some INSTIs do indeed have a direct impact on adipocytes: DTG and (to a lesser extent) RAL increased ECM accumulation and adipocyte hypertrophy. Despite the increase in adipogenesis, INSTIs also promote oxidative stress and insulin resistance (123). The mechanisms involved are being studied but one can reasonably hypothesize that adipocyte hypertrophy in the abdominal SCAT of patients on INSTIs compensates for the decreases in beige AT and energy expenditure. Interestingly, a recent study showed that the brown AT transcription factor PRDM16 might protect against the development of fibrosis in WAT (145). Furthermore, TGF-β inhibits the beiging of adipocyte precursors (146). A decline in beiging might therefore also be involved in the fibrosis observed in the AT of INSTI-treated patients. These associations highlight the need to determine whether people living with HIV (PLWH) are at risk of cardiometabolic disease as a result of reduced beiging capacity in WAT; this might constitute a novel mechanism for metabolic dysregulation in this population.

Another hypothesis relates to the effect of INSTIs on food intake. It has been shown that DTG inhibits the binding of α-melanocyte-stimulating hormone (αMSH) and melanocortin 4 receptor (MC4R) in the hypothalamus; this might promote hyperphagia (147, 148). However, the serum DTG levels in patients are much lower than those required for hyperphagia (149).

In the context of obesity, elevated fibrosis is mainly due to periadipocyte fibrosis; the latter has been linked to metabolic disorders (60) and was observed in the AT of INSTI-treated macaques and patients. These alterations might result from increased oxidative stress. Indeed, lipohypertrophy in PLWH has previously been linked to mitochondrial toxicity in AT (150). Accordingly, we found that DTG and RAL promoted ROS production and mitochondrial dysfunction. We hypothesized that in the context of HIV infection and stressed adipocytes in a profibrotic environment (due to the impact of HIV, obesity, or antiretrovirals other than INSTIs), INSTIs might alleviate adipocyte stress (thereby favoring hypertrophy/expansion) but might also increase oxidative stress, reduce metabolic flexibility, and accentuate insulin resistance.

#### Systemic Effects

After secretion by AT, the hormones adiponectin and leptin regulate key pathways in lipid and glucose metabolism (151). Adiponectin has a potent anti-inflammatory and anti-atherogenic action, and a decrease in adiponectin levels is correlated with the development of insulin resistance (152). Adiponectin also has an important role in lipid metabolism by increasing fatty acid oxidation in skeletal muscle and in the liver and thus lowering circulating FFA levels (153). In contrast, leptin regulates the energy balance by inhibiting hunger but also has major effects on insulin sensitivity and inflammation (154). In accordance with the observed alterations in AT function, circulating levels of adiponectin and leptin are low in both ART-treated and treatment-naive patients (155). Furthermore, some studies have shown that DTG and RAL lower circulating adiponectin levels (156–160). Recently, it has been suggested that the adiponectin/leptin ratio is a marker of AT dysfunction; threshold values for cardiometabolic risk have been determined (161, 162) and could therefore be explored in ART-experienced HIV-infected patients.

Metabolite profiles are altered not only in plasma/serum but also in a variety of other biological fluids. In cerebrospinal fluid, alterations in energy metabolites were found to be correlated with neurocognitive impairments and increased inflammation in ART-experienced HIV-infected individuals. These alterations overlap with the metabolic alterations seen in older HIV-negative individuals and suggest that aging is accelerated by exposure to ART (163).

### Immune Changes

#### AT-Resident Immune Cells

Studies of samples collected from HIV-infected people (mostly ART patients) and HIV-negative controls are difficult to interpret because of large intergroup differences in infectious, metabolic and treatment-related parameters. In this respect, nonhuman primates are interesting models for determining the effect of HIV in the presence or absence of ART, and provide a more controlled experimental setting with regard to the individuals’ infectious and metabolic histories. Regardless of the model and the stage of infection considered, mRNA/protein quantification (on supernatants or microscopy) and flow cytometry studies (102, 103, 164) have shown that chronic HIV/SIV infection is associated with a shift towards a pro-inflammatory immune profile in AT. In humans, levels of adipose-macrophage-derived cytokine (IL-12, IL-6, IL-8, and MCP-1) are higher in HIV-infected individuals than in noninfected individuals. In cynomolgus macaques, the overall macrophage count in AT was similar in HIV-infected and noninfected individuals but the former group had a higher proportion of non-M2 macrophages (based on the expression of CD206 and CD163 as M2 markers) (102). Changes in the T cell compartment have been reviewed in detail (165). The most important change is a decrease in the CD4/CD8 ratio, with an accumulation of CD8 T cells during chronic untreated SIV infections (101, 102). Research on the CD4 T cell fraction (166, 167) in both humans and nonhuman primates revealed an AT-specific phenotype, whereas differences between HIV/SIV+ and HIV/SIV noninfected animals were limited. AT-resident CD4 T cells are characterized by higher expression levels of PD-1 and CD57 and higher proportions of effector memory (Tem) and CD45RA+ Tem (Temra) - suggesting a more activated/exhausted profile. High expression of CD69 on AT-resident T cells has also been reported (166, 167), although this marker is associated with resident memory cell differentiation and/or activation. The Treg fraction (a seminal immunoregulatory subset) was reportedly prominent in the AT in some mouse models (31, 168). In contrast, baseline Foxp3 expression was low in both HIV-infected and noninfected people, even though the proportion of Tregs was slightly higher in SIV-positive animals (166).

Overall, chronic HIV infection appears to have the same effects on AT as obesity (a shift towards M1 macrophages and CD8 T cell accumulation), albeit to a lesser extent. Data on the modulation of other immune cell fractions present in AT during HIV infection (in the presence or absence of ART) are lacking. Likewise, HIV-related changes in the status of neutrophils, CD4 Th1 T cells, and type 1 innate lymphoid cells as effectors or partners in pro-inflammatory immune responses have not yet been studied. However, this knowledge may be needed to fully understand the complex crosstalk between immune and metabolic cells in AT and to further evaluate local inflammation in AT during HIV infection.

#### Inflammation

The pro-inflammatory cytokine profile of AT in HIV/SIV-infected individuals vs. noninfected individuals has been characterized by detecting proteins or mRNA in AT lysates or supernatants of AT fractions. Although the studies differed with regard to the experimental methods and the cytokines assayed (91, 101, 167), all found a greater pro-inflammatory potential (with IL-2, IL-7, IL-15, CCL19, and TGF-β) in AT collected from HIV/SIV-infected individuals vs. noninfected individuals. Koethe et al. also contributed important insights into the transcriptional upregulation of genes coding for chemokines, chemokine receptors, and Toll-like receptors (167). Taken as a whole, these data suggest that chronic infection induces a pro-inflammatory profile in AT. Importantly, all the studies performed on human samples were limited by high inter-individual heterogeneity, which presumably reflects differences in metabolic and infectious histories. Important, Couturier et al. compared the AT cytokine secretion profiles in ART-experienced patients and treatment-naive patients; ART appeared to be associated with a less inflammatory profile in the AT (101).

The net impact of ART is thus obviously highly beneficial because it controls the HIV infection. ART reduces the viral load, restores (at least partially) the CD4 T cell count, and dampens inflammatory processes. This benefit is also observed within the AT, with regard to inflammatory cytokines. However, ART induces metabolic side effects (which are highly dependent on the combination used) that change the nature of the chronic inflammation associated with a controlled HIV infection. As described in this section, the ARVs’ side effects affect ASCs and/or adipocytes and can induce oxidative stress, ECM remodeling (with a consequential reduction in the metabolic responsiveness of AT), and insulin resistance. Importantly, recent studies have also evidenced a direct impact of ARVs on immune cell functions (169). DTG and elvitegravir reduce the oxidative and proliferative activities of peripheral blood CD4 T cells and thus induce a “stress” immune response with strong production of TNF but no polyfunctional cytokine responses. Based on these data, the ARV’s impact on immune activity (and not just metabolic activity) in AT should also be investigated.

#### Oxidative Stress, Endothelial Dysfunction, and the Renin-Angiotensin-Aldosterone System

In the context of AT disease such as obesity, vascular damage, including endothelial dysfunction, has been observed, along with oxidative stress. Increased oxidative stress develops in obese AT as a result of multiple factors: dysfunctional adipocytes in response to nutrient overload (hypertrophy, increased mitochondrial oxidation, increased levels of reactive oxygen species (ROS)), AT hypoxia, increased pro-oxidant cytokines production (such as TNF-α) by AT and increased release of FFAs. These parameters contribute to systemic oxidative stress and inflammation (170). Obesity is also associated with vascular remodeling and fibrosis in both VAT and SCAT (171). This vascular remodeling is associated with inflammation characterized by macrophage infiltration especially in SCAT and has been associated with systemic endothelial dysfunction and insulin resistance in obese patients (172).

The renin-angiotensin system (RAS) plays an important pathophysiological role. The production of RAS components by adipocytes is exacerbated during obesity, contributing to the systemic RAS which then affects the cardiovascular system (171). The up-regulation of adipose RAS, and especially the increase of angiotensin II production, promotes inflammation through increased chemokine production, oxidative stress and fibrosis (171). Conversely, blocking or preventing RAS activation in AT can reduce oxidative stress, improve insulin resistance and reduce inflammation (173, 174). Interactions between the RAS and HIV infection have been described, and could contribute to AT inflammation, but also to the onset of metabolic syndrome and hypertension in PLWH (175, 176).

#### Insulin Resistance and Lipotoxicity

AT dysfunction, as seen in obesity and diabetes, is generally associated with insulin resistance, a metabolic disorder affecting multiple tissues. The proposed mechanisms involved in insulin resistance include both whole body aspects, such as inflammation and metabolic inflexibility; as well as cellular phenomena, such as lipotoxicity, ER stress, and mitochondrial dysfunction. Lipotoxicity, as a result of increased lipolysis in AT and release of FFAs, is a type of cellular stress induced by the accumulation of lipid intermediates such as diacylglycerols (DAGs), ceramides, and triglycerides that facilitate the development of insulin resistance and ectopic fat deposition in muscle, liver, and adipose tissue together with a pro-inflammatory response (177). Lipotoxicity participates to the enhanced cardiometabolic risk observed in PLWH (178, 179).

## Comorbidities Associated With Low-Grade Inflammation

In the general population, a number of age-related comorbidities (including cardiometabolic diseases, neurocognitive impairments, cancer, and frailty) are associated with a state of low-grade chronic inflammation. Circulating levels of several cytokines and inflammatory markers [such as IL-6 and C-reactive protein (CRP)] rise with age and might trigger these comorbidities. AT is the main source of circulating IL-6, and the cytokine triggers the synthesis of CRP in the liver. Accordingly, obesity is associated with low-grade, chronic inflammation. Hence, an increase in the mass of AT might contribute to these comorbidities by enhancing low-grade inflammation.

Several studies have evidenced a state of low-grade inflammation in ageing HIV-infected patients who are well controlled by ART. This level of inflammation is greater than in non-infected subjects (180, 181) and is thought to be involved in the elevated prevalence of certain comorbidities. The inflammation might result from HIV-related factors, such as the presence of the virus inside reservoirs, greater gut permeability, dysbiosis, an effect on gut-associated lymphoid tissue, and coinfections (which are nevertheless observed in ART-controlled patients). AT might be a key factor - first as an HIV reservoir and then as a source of inflammatory markers in the context of ART-related fat mass gain.

Some patients treated with thymidine NRTIs present persistent fat alterations several years after treatment discontinuation. In the AGEhIV cohort, long-term-infected, ART-controlled patients had a greater waist circumference, a lower hip circumference, and a greater incidence of hypertension than HIV-negative people (180). In the COCOMO study, cumulative exposure to stavudine, zidovudine or didanosine was associated with elevated amounts of VAT, lower amounts of SCAT, and a higher risk of hypertension and dyslipidemia (182).

**Figure 4:**
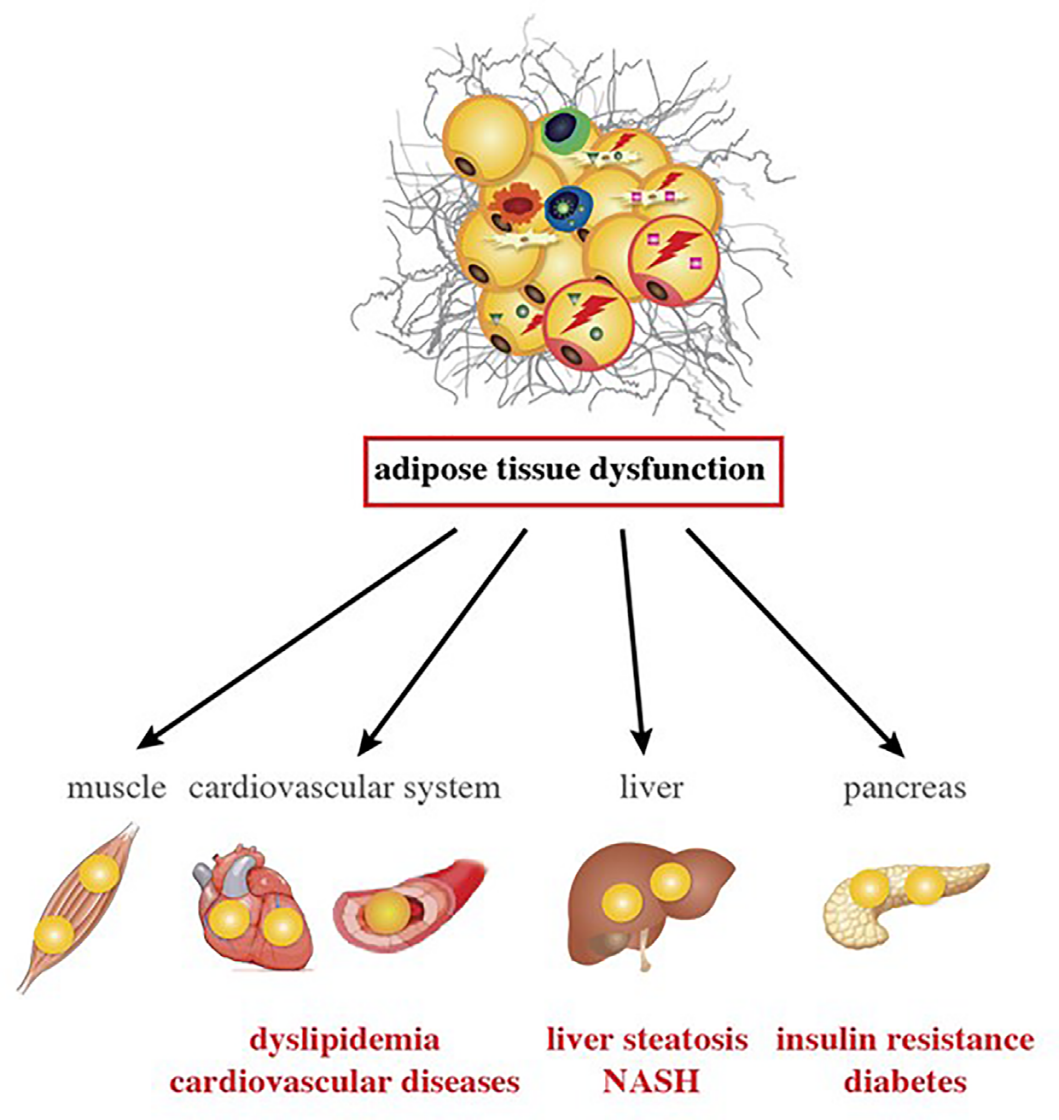
Inflammation changes in AT and are associated with comorbidities in ART-experienced, HIV-infected patients. AT alterations with VAT expansion, local and systemic inflammation, and gut dysfunction promote lipotoxicity in ART-experienced, HIV-infected people. Lipids accumulate in metabolic organs other than the AT, such as muscle, heart, blood vessels, liver, and pancreas. Lipotoxicity and inflammation might contribute to the development of atherogenic dyslipidemia, cardiovascular diseases, insulin resistance, diabetes, liver steatosis and NASH in the context of HIV infection and ART.

Trunk fat accumulation is one facet of the broader condition of ectopic lipid deposition in HIV-infected patients; accumulation is also observed in the liver, epicardial tissue, and skeletal muscle. In this situation (also referred to as “metabolically unhealthy obesity”, due to its association with cardiometabolic outcomes), the storage capacity of SCAT (the largest WAT depot) is limited, and further calory overload leads to fat accumulation in ectopic tissues (e.g., liver, skeletal muscle, and heart) and in VAT depots - an event commonly referred to as “lipotoxicity”. Excessive ectopic lipid accumulation leads to local inflammation and insulin resistance (42). The clinical consequences of ectopic fat deposition are summarized in **Figure 4**.

### Atherosclerotic Cardiovascular Disease

Epicardial fat is physiologically similar to visceral fat and is located close to the coronary arteries. It might therefore have a major role in the development of coronary atherosclerosis (183, 184). Groups of adipocytes are close to the right ventricular myocardium and might release fatty acids to fuel the cardiomyocytes. Epicardial fat also releases adipokines, cytokines, and vasoactive products that regulate coronary artery tone. In obese patients with several CV risk factors, epicardial fat around the coronary arteries releases pro-inflammatory cytokines and presents a high macrophage count and high levels of TNF-α, IL-6, and IL-8 (185). It has been suggested that the greater volume of epicardial fat in obese subjects contributes to the elevated CV risk (186).

Several studies of ART-controlled patients have evidenced greater volumes of epicardial fat; along with traditional risk factors for atherosclerosis and the duration of ART, the amount of epicardial fat is associated with the amount of VAT and with insulin resistance. In HIV-infected patients, the epicardial fat volume is related to endothelial dysfunction and the progression of carotid atherosclerosis (187–189).

More recently, the overall fat gain observed in some patients raises the question of whether treatment with INSTIs influences cardiometabolic outcomes. At present, there is no indication of an elevated CV risk for this class of ART molecules, although long-term follow-up data (i.e. for at least 5 years) are missing.

### Metabolic Liver Diseases

Liver steatosis corresponds to another type of ectopic fat deposition. An elevated liver fat content has been reported in patients with lipoatrophy and in those with central obesity - two situations associated with systemic low-grade inflammation. In HIV-mono-infected patients, the prevalence of steatosis is relatively high, relative to the body mass index (BMI) (30-35%) (190). In some people, simple steatosis can progress to a more worrying situation: non-alcoholic steatohepatitis (NASH), with liver inflammation and often progressive fibrosis. Severe fibrosis (cirrhosis) and progression to hepatocarcinoma are life-threatening.

Several studies have highlighted the strong relationship between trunk fat accumulation and steatosis or fibrosis in HIV-infected subjects. In a matched cohort of 468 HIV-mono-infected patients the prevalence of significant liver fibrosis (≥Fibroscan grade 2) was 25.1% in patients with metabolic syndrome and 7.9% in those without. In a multivariate analysis, obesity and insulin resistance were independently associated with advanced fibrosis (≥Fibroscan grade 3). Leptin and soluble (s) CD163 were strongly associated with fibrosis/cirrhosis, whereas HIV parameters and ART were not. Thus, AT and macrophage activation might be key players in the development of liver fibrosis in HIV-infected patients (191). In a study of 62 HIV-infected adults with elevated aminotransferase levels, insulin resistance, obesity, and the presence of either of two minor alleles in the *PNPLA3* gene were significantly associated with an elevated risk of NASH and fibrosis. NASH and/or fibrosis were not associated with the duration of HIV infection or ART (192).

### Insulin Resistance and Diabetes

In the APROCO cohort of HIV-infected patients followed up for 10 years after the initiation of first-generation PIs, the incidence diabetes peaked in 1999-2000, and was linked to treatment with indinavir, stavudine and didanosine. As expected, diabetes was associated with age and adiposity. The main risk factor was the waist-to-hip ratio (hazard ratio: 3.9), which highlighted the key role of truncal obesity (193). In a longer-term study of 352 patients from the cohort, the prevalence of diabetes was 11%, and the prevalence of atherogenic dyslipidemia (a high triglyceride/high-density lipoprotein ratio, observed mainly in diabetic subjects) was 9%. Diabetes and atherogenic dyslipidemia were associated with elevated levels of systemic markers of oxidative stress and inflammation (194).

The more recent use of INSTIs has resulted in overall fat gain in some patients. While blood lipid levels are generally lower, the outcome for insulin resistance is less clear. We have observed elevated insulin resistance in well-controlled, aging patients switched from a PI-containing regimen to RAL/etravirine; this is probably linked to INSTI-related weight gain (195). Regarding diabetes, 722 of the 22,884 eligible individuals (3%) in the USA/Canadian NA-ACCORD study of ART-initiators developed this condition. Persons initiating INSTIs + NNRTIs had the same incident risk of diabetes as those initiating PIs+ NNRTIs. This effect was most pronounced for RAL. The association between INSTI and diabetes was attenuated by accounting for 12-month weight gain. Initiating combination ART regimens with INSTIs or PIs + NNRTIs may confer a greater risk of diabetes; this is probably mediated by weight gain (196). In the French DAT’AIDS study, 265 of the 19,462 HIV-infected patients initiating ART developed diabetes. Multivariate and survival analyses did not highlight an increase in new-onset diabetes in patients undergoing combination ART with an INSTI as a third agent (relative to an NNRTI or a PI). BMI, age, African or Hispanic ethnicity, arterial hypertension, and AIDS were associated with a higher incidence of diabetes (197).

Hence, it is currently not clear whether INSTIs have an additional effect on the incidence of diabetes, other than that related to weight gain. However, clinicians should be aware of this possible metabolic comorbidity - particularly in older patients and in patients with a high BMI.

Relative to non-infected subjects, HIV-infected patients present a higher prevalence of age-related comorbidities associated with a higher level of systemic inflammation. This might result from a number of factors related to HIV infection and coinfections (with hepatitis C virus or CMV) but also modifiable risk factors such as smoking, alcohol abuse and substance abuse (181, 198). Furthermore, adiposity and particularly truncal fat accumulation are associated with low-grade inflammation and comorbidities resulting from greater ectopic fat deposition.

## Therapeutic Strategies

Here, the goal of treatment (in addition to ART) is to decrease inflammation, immune activation, and thus the related comorbidities. Even though these strategies do not specifically address AT, they might act on this tissue in addition to other organs. As observed for obesity-related cardiometabolic complications in the general population, weight reduction has been consistently associated with lower AT inflammation and results in better cardiovascular, hepatic and metabolic outcomes (i.e. a lower incidence of NAFLD, insulin resistance, and diabetes) (199). This approach is relevant in overweight or obese HIV-infected patients with central fat accumulation. Furthermore, regular exercise is an excellent strategy for reducing inflammation and cardiometabolic complications (199).

Other strategies aiming at reducing systemic inflammation to reduce comorbidities have been tested in HIV-infected patients and are reviewed elsewhere (198). The lasting association between inflammation and atherosclerosis has prompted significant interest in strategies for inhibiting inflammatory proteins upstream of CRP - particularly IL-6 and IL-1β. The strongest evidence to date in favor of preventing CVD by reducing inflammation comes from the CANTOS study using canakinumab, inhibiting IL-1β, albeit in HIV-negative participants (200). In HIV-infected patients, Hsue et al. recently demonstrated that IL-1β inhibition with canakinumab reduced atherosclerotic inflammation (201) but was not associated with other improvements. In a phase II trial, HIV-infected patients at an increased risk of atherosclerotic events were treated with low-dose methotrexate or placebo for 24 weeks (202). Low-dose methotrexate had no effect on the primary endpoint (brachial artery flow-mediated dilation) or levels of various inflammatory biomarkers, and the trial was stopped prematurely. The potential effect of the Janus kinase inhibitor ruxolitinib was evaluated in an open-label trial in HIV-positive patients (Marcolin VC, unpublished data). At week 5, the sCD14 level was significantly lower in the ruxolitinib arm than in the ART-alone arm but the IL-6 level was not. Since D-dimer elevation is linked to the pro-inflammatory profile reported in PLWH, it has been suggested that anticoagulant drugs may be of value in treating the pro-coagulant state. Orally administered edoxaban (a direct factor Xa inhibitor) was tested against placebo in the TACTICAL-HIV trial (Baker JV, unpublished data). However, four months of edoxaban treatment did not lead to lower levels of biomarkers associated with inflammation or monocyte activation.

The anti-inflammatory activity of 3-hydroxymethyl-3-methylglutaryl coenzyme A reductase inhibitors (i.e. statins) is well documented (203), and several clinical trials have assessed the benefits of these drugs on surrogate markers of CVD (along with LDL-lowering activity) in ART-suppressed HIV-infected patients. In the SATURN-HIV trial, rosuvastatin (10 mg/day) was significantly associated with low levels of sCD14, with a fall of approximately 10% at 48 weeks (204). Lower levels of several markers of vascular inflammation were also observed. In the INTREPID trial, similar results were observed for pitavastatin but not for pravastatin (205). It is now necessary to determine whether the statin-induced reduction of inflammatory/immune activation and cardiovascular biomarkers translates into a reduction in morbidity and mortality in HIV-infected patients. The ongoing REPRIEVE study is addressing this question (206).

Taken as a whole, strategies designed to decrease systemic inflammation/immune activation in HIV-infected patients have not produced a clear reduction in comorbidities. Again, combinations of simple lifestyle modifications (e.g. exercise and weight loss) with decreased AT inflammation have been validated for reducing the incidence of cardiovascular events, diabetes, and NAFLD.

## Conclusion

AT is targeted by both HIV infection and ART. HIV targets AT both directly (direct infection of AT CD4 T cells) and indirectly (viral protein release, inflammatory signals, and gut disruption). Schematically, ART impacts adipogenesis, adipocyte homeostasis, and ECM remodeling (including fibrosis), whereas viral persistence directly impacts the metabolic and immune functions of AT immune cells. In a chronic ART-controlled HIV infection, the direct impact of HIV *per se* is less severe than that of ART. Indeed, ART efficiently reduces the viral load and the various immune defects associated with the early stage of infection. However, restoration of the immune system is only partial, and the persisting damage inflicted on the immune system during the early stages of infection might contribute to AT inflammation. Given the close links between the gut and AT, the latter’s functions may also be indirectly impacted by the massive CD4 T cell depletion observed in the gut in the early stages of infection. Subsequently, AT’s endocrine activity and its release of metabolites will increase systemic immune activation and inflammation. Furthermore, ART reduces the viral load to undetectable levels in the circulation blood but does not eradicate the virus in reservoir sites such as AT.

In the longer term, the various direct toxic effects of ARVs might drive low-grade inflammation in HIV-infected patients. These effects are highly dependent on the classes/combinations of ARV used, that variously target ASCs and/or adipocytes, and can induce oxidative stress, ECM remodeling (with a consequential reduction in the metabolic responsiveness of AT), and insulin resistance (125). Ongoing research is also evaluating the direct impact of ARVs on immune cells. Given the crucial, pleiotropic, metabolic role of AT and the complex, intricate dialogue between AT and HIV-related or ART-related parameters, it is still very difficult to evaluate the contribution to systemic inflammation of AT in general and HIV-related changes in AT in particular. These contributions might also depend strongly on metabolic alterations and comorbidities present at the start of the infection.

## Author Contributions

All authors contributed to the article and approved the submitted version.

## Funding

The research was funded by the French National Research Agency for HIV and Viral Hepatitis (ANRS), the French National Research Agency (reference: RHU CARMMA ANR-15-RHUS-0003), Sidaction, DIM OneHealth, Fondation Dormeur, and Gilead.

## Conflict of Interest

The authors declare that the research was conducted in the absence of any commercial or financial relationships that could be construed as a potential conflict of interest.
